# Extracellular Vesicles in Cardiac Amyloidosis: From Pathogenesis to Clinical Applications

**DOI:** 10.3390/diagnostics16030430

**Published:** 2026-02-01

**Authors:** Ashot Batikyan, Donclair Brown, Zainab Elahmadi, Joo Hee Park, Ashwin Ragupathi, Petras Lohana, Panagiotis Zoumpourlis, Priyansh Shah, Modak Vishakha, Martin Mcintosh, Michail Kladas, Priyanka Gokulnath, Michail Spanos

**Affiliations:** 1 Albert Einstein College of Medicine, North Central Bronx Hospital, Bronx, NY 10467, USA; ashotbatikyan14@gmail.com (A.B.); elahmadz@nychhc.org (Z.E.); yourstrulyjoohee@gmail.com (J.H.P.); ashwinragu1996@gmail.com (A.R.); drpetraslohana@gmail.com (P.L.); martin.n.mcintosh@gmail.com (M.M.); kladasm@nychhc.org (M.K.); 2 Montefiore Medical Center, Albert Einstein College of Medicine, Bronx, NY 10461, USA; donclair.brown@gmail.com; 3 Albert Einstein College of Medicine, Jacobi Medical Center, Bronx, NY 10461, USA; zoumpoup@nychhc.org (P.Z.); drpriyanshshah@gmail.com (P.S.); vishakhaa.modak@gmail.com (M.V.); 4 Cardiovascular Research Center, Massachusetts General Hospital & Harvard Medical School, Boston, MA 02114, USA

**Keywords:** cardiac amyloidosis, extracellular vesicles, biomarkers, transthyretin, amyloid cardiomyopathy, precision medicine

## Abstract

Cardiac amyloidosis is an infiltrative cardiomyopathy caused by extracellular deposition of misfolded proteins, most commonly immunoglobulin light chains (AL) or transthyretin (ATTR), with rarer forms occurring less frequently. AL amyloidosis arises from plasma cell-derived light chains and typically follows an aggressive clinical course, whereas ATTR amyloidosis results from misfolded wild-type or variant transthyretin and progresses more indolently. Extracellular vesicles (EVs) have recently been recognized as mediators of amyloid propagation, inflammation, and myocardial remodeling, particularly at later stages of disease. Despite growing evidence, no comprehensive reviews have focused on this relationship. We conducted a structured narrative review (PubMed and Scopus, 2020–2025) following Preferred Reporting Items for Systematic reviews and Meta-Analyses extension for Scoping Reviews (PRISMA-ScR) guidelines to synthesize emerging data. EVs act as scaffolds for transthyretin and serum amyloid A aggregation and carry disease-specific protein and RNA cargo detectable in blood and urine. Experimental models also demonstrate EV-mediated transport of serum amyloid A under conditions of cardiac stress, representing a reactive amyloidogenic pathway rather than a common cause of human cardiac amyloidosis. Preclinical studies show regenerative and anti-fibrotic effects of stem-cell-derived EVs, and early clinical trials demonstrate the feasibility of EV-based cardiac therapy. While methodological and translational challenges persist, EVs represent promising diagnostic and therapeutic tools that could transform the precision management of cardiac amyloidosis.

## 1. Introduction

Amyloidosis is a condition characterized by extracellular deposition of amyloid fibrils derived from various precursor proteins that self-assemble into highly ordered abnormal cross β-sheet conformations [1]. In cardiac amyloidosis, amyloid fibrils accumulate within the heart’s extracellular space, most commonly due to immunoglobulin light-chain amyloidosis (AL) or transthyretin amyloidosis (ATTR). Accordingly, this review focuses on AL and ATTR cardiac amyloidosis; serum amyloid A (SAA)-related amyloidogenesis is discussed as a distinct reactive/secondary mechanism, since classical AA amyloidosis only rarely involves the heart. In general, this infiltrative process leads to myocardial stiffening with diastolic dysfunction (often manifesting as restrictive physiology), progressive heart failure, and an arrhythmia burden that is predominantly atrial—driven in part by atrial amyloid cardiomyopathy and commonly presenting as atrial fibrillation—whereas ventricular arrhythmias are less frequent [2,3,4].

Immunoglobulin light-chain (AL) amyloidosis results from a clonal plasma cell disorder producing misfolded light chains that deposit in the myocardium, typically leading to rapid disease progression and high early mortality if untreated [3]. In contrast, transthyretin amyloidosis (ATTR) is caused by misfolding of transthyretin, either due to age-related instability of wild-type TTR (ATTRwt) or pathogenic gene variants (ATTRv), and generally follows a more indolent clinical course [2]. Therapeutic strategies also differ substantially: AL amyloidosis relies on plasma cell-directed therapies, whereas ATTR management includes TTR stabilizers and gene-silencing approaches targeting hepatic TTR production [5]. Throughout this review, we organize available evidence by amyloid subtype whenever possible. Direct EV-mediated amyloid seeding has been demonstrated most clearly in hereditary ATTR (ATTRv), whereas EV roles in AL and wild-type ATTR (ATTRwt) remain less defined and are often inferred from broader EV biology (e.g., inflammation, fibrosis, endothelial dysfunction, and remodeling). Where subtype-specific evidence is unavailable, we explicitly note these gaps.

Extracellular vesicles (EVs) are lipid bilayer-bound particles released by cells that mediate intercellular communication [6]. EVs carry a diverse cargo of proteins, lipids, mRNAs, microRNAs (miRs), and even DNA, reflecting the physiological or pathological state of their parent cells [7]. These EVs influence gene expression, cell phenotype, and molecular pathways [8]. Three main EV subtypes are recognized: exosomes (30–150 nm vesicles of endosomal origin), microvesicles (100–1000 nm, shed from the plasma membrane), and apoptotic bodies (500–2000 nm, released from dying cells) [9]. Extracellular vesicles (EVs) and the microRNAs (miRs) are crucial in the development and progression of cardiovascular diseases (CVD) [10]. Functionally, EV-associated miRs can be internalized by recipient cells where they post-transcriptionally regulate target mRNAs, reshaping signaling pathways involved in inflammation, fibrosis, hypertrophy, endothelial dysfunction, and electrophysiologic remodeling [11,12]. EVs not only impact inflammation, coagulation, and angiogenesis, but can also serve as biomarkers for diagnosing and treating CVD [13]. In this context, plasma EV transcriptomes have been shown to function as a “liquid biopsy” in acute heart failure, with EV-derived long RNA signatures distinguishing HFpEF from HFrEF and reflecting distinct underlying biology across subtypes [14]. Given these roles, EVs are being explored both as pathogenic contributors to disease and as novel diagnostic and therapeutic tools.

This review provides an updated synthesis (2020–2025) of the roles of EVs in cardiac amyloidosis, spanning from disease pathogenesis to clinical applications. We discuss new evidence, highlighting recent advances in EV-based biomarkers and therapies. Specifically, we examine: (1) EV contributions to amyloid pathogenesis, (2) ongoing clinical trials of EV-based therapies in cardiovascular or amyloid diseases, and (3) emerging therapeutic applications and their limitations. Finally, we address gaps and future directions, particularly EVs as biomarkers and precision therapeutics in cardiac amyloidosis.

## 2. Methods

This narrative review followed structured methods consistent with PRISMA-ScR and International Society for Extracellular Vesicles (ISEV) 2023 recommendations for extracellular vesicle (EV) research. A comprehensive literature search was performed in PubMed, Scopus, and Web of Science for English-language articles published between January 2020 and September 2025 using combinations of the following keywords: extracellular vesicles, exosomes, cardiac amyloidosis, transthyretin amyloidosis, light-chain amyloidosis, EV biomarkers, and EV therapy. Reference lists of relevant papers were also manually screened. Eligible studies included: (1) experimental studies (in vitro, animal, and human) investigating EV mechanisms in amyloidosis or cardiac remodeling, (2) clinical studies or trials evaluating EVs as biomarkers or therapeutic agents in cardiovascular or amyloid diseases, and (3) recent reviews and consensus statements that clarified EV nomenclature, isolation, and quantification.

Studies were excluded if they lacked primary data on EVs, were not peer-reviewed, or duplicated previous publications. Data on study design, EV source and isolation method, sample size, key molecular findings, and clinical outcomes were extracted. Findings were synthesized thematically across three domains: (1) EV-mediated pathogenic mechanisms in amyloidosis; (2) therapeutic applications of EVs; and (3) clinical implications and future directions. Where available, clinical trials were verified through the ClinicalTrials.gov registry.

## 3. Pathogenic Mechanisms of EVs in Cardiac Amyloidosis

Pathogenesis of cardiac amyloidosis involves not only the overproduction or mutation of amyloidogenic proteins but also the local factors that influence amyloid assembly and deposition. Emerging evidence implicates EVs as active participants in amyloid protein aggregation and tissue deposition. EV membranes can serve as scaffolds or catalysts for amyloid fibril formation, and EV cargo (proteins or RNA) can modulate the cellular environment to favor amyloid accumulation [15].

### 3.1. ATTR Amyloidosis: EV-Associated TTR Aggregation and Deposition

One striking example is hereditary ATTR amyloidosis. A study by Yamaguchi et al. demonstrated that mutant TTR (Val30Met) is carried on circulating EVs and aggregates preferentially on EV membranes, which then promote deposition of TTR amyloid in recipient cells [16]. In cell culture, the presence of serum-derived EVs markedly enhanced the attachment of TTR aggregates to cells [16]. Moreover, patients with ATTRv amyloidosis had a lower abundance of TTR carried in their EVs compared to healthy individuals, consistent with the hypothesis that EV-bound TTR is being deposited in tissues [16]. These findings imply that EV surface molecules may act as nucleation sites for amyloid and could direct amyloid proteins to specific organs. Indeed, it has been suggested that certain EV membrane proteins guide amyloidogenic proteins to particular tissues, dictating where amyloid deposits form [17]. Thus, EVs can actively influence where and how amyloid builds up in the heart and other organs. Investigating TTR aggregation and the uptake of EVs by recipient cells in affected tissues is essential to understanding the role EVs play in the pathology of familial ATTR. Furthermore, further research is needed to determine whether similar EV-mediated nucleation occurs in ATTRwt cardiomyopathy, and direct mechanistic evidence for EV-driven amyloid seeding in AL cardiac amyloidosis is currently lacking.

### 3.2. Reactive Cardiac Amyloidogenesis via EV-SAA3 After Myocardial Injury (Non-AL/ATTR Mechanism)

Another mechanistic link between EVs and cardiac amyloidogenesis comes from studies of inflammation and tissue injury. Myocardial infarction (MI) can create a pro-amyloidogenic milieu in the heart. In a 2025 study, Cimini et al. demonstrated that following an MI in mice, a subset of cardiac stromal cells (characterized by the glycoprotein podoplanin) produced exosomes highly enriched in serum amyloid A3 (SAA3). These SAA3-laden exosomes triggered a Toll-like receptor 2 (TLR2) response in macrophages, inducing a feed-forward loop of SAA3 overproduction and amyloid deposition in the infarcted heart. The result was an SAA3 amyloidosis in the injured myocardium, which exacerbated inflammation and worsened post-MI left ventricular function [18]. Notably, blocking SAA3 aggregation with a specialized retro-inverso D-peptide halted amyloid fibril formation and improved cardiac function in this model [18]. This work reveals an EV-driven mechanism of reactive amyloid deposition: stress-induced EV cargo (in this case SAA3) can initiate local amyloid formation in the heart. Importantly, this model reflects stress-induced, localized SAA3 deposition after myocardial injury rather than the typical clinical pattern of systemic AA amyloidosis, which seldom presents as primary cardiac amyloidosis. It also highlights how EVs act as intermediaries between injured stromal cells and immune cells, propagating signals that lead to amyloid fibril accumulation.

### 3.3. Evidence Gaps in AL and ATTRwt

Beyond TTR and SAA3, EVs may influence other amyloidogenic processes relevant to the cardiovascular system. For example, medin amyloidosis—an age-associated amyloid deposition in the aorta, has recently been linked to EV activity. Senescent vascular smooth muscle cells were shown to secrete more small EVs due to upregulation of sphingomyelinase (SMPD3), and these EVs accelerated aggregation of medin peptides in the extracellular matrix [19]. This suggests a general principle that cell stress or aging can increase EV release of amyloidogenic factors. Additionally, EVs might facilitate the spread of misfolded protein aggregates between cells, analogous to prion-like propagation observed in neurodegenerative amyloidoses. In the brain, for instance, exosomes can carry amyloid-β or tau seeds and contribute to Alzheimer’s disease pathology [20]. By extension, EV-mediated intercellular transfer of amyloidogenic species could be relevant to cardiac amyloidosis; however, while EV-bound TTR aggregation has been shown in ATTRv models, direct evidence for EV-mediated trafficking or seeding of immunoglobulin light chains in AL, or amyloidogenic TTR species in ATTRwt cardiomyopathy, remains unavailable.

In summary, EVs participate in the pathogenesis of cardiac amyloidosis through multiple mechanisms: (1) serving as scaffolds that concentrate and nucleate amyloid fibrils on their surfaces; (2) shuttling pathogenic proteins (SAA3, mutant TTR, etc.) and enhancing their deposition in target tissues; and (3) modulating immune and inflammatory pathways that indirectly foster amyloid buildup, including EV-triggered activation of innate immune receptors on macrophages (e.g., TLR2), which can amplify inflammatory feed-forward loops and accelerate amyloid deposition and post-injury remodeling. Beyond subtype-specific amyloidogenic effects, EVs likely contribute to shared downstream pathways in both AL and ATTR, including macrophage activation, endothelial dysfunction, microvascular inflammation, and pro-fibrotic remodeling; however, the extent to which these EV signatures differ by subtype has not yet been systematically defined.

## 4. Clinical Applications of EVs in Cardiac Amyloidosis

The unique properties of EVs have sparked interest in exploiting them for therapy (Table 1). Because EVs are endogenous nanocarriers, they offer several advantages over synthetic drug delivery systems: low immunogenicity, inherent biocompatibility, ability to cross biological barriers, and protection of cargo from degradation [21]. Two broad therapeutic approaches have emerged: (1) EVs as drugs, harnessing the native functions of EVs from certain cell types to counter disease processes, and (2) EVs as delivery vehicles—customizing EVs to carry drugs (such as small RNAs, peptides, or small molecules) to target tissues. In the context of cardiac amyloidosis, because AL and ATTR differ in the source of amyloidogenic protein (plasma cell-derived light chains vs. hepatic TTR), EV-based therapeutic strategies may be best considered in two parallel tracks: (i) myocardial repair or anti-fibrotic EV therapies applicable to both subtypes, and (ii) subtype-specific upstream targeting (plasma cell dyscrasia in AL vs. hepatic TTR production in ATTR) [22]. Regeneration or restoration of cardiac tissue damaged by amyloid is an area in which EVs show great promise. Multiple preclinical studies suggest that stem- and progenitor-cell-derived EVs can reproduce several cardioprotective paracrine effects attributed to cell therapy, and may offer practical advantages as acellular biologics (e.g., simplified handling and reduced concerns related to cell engraftment), although comparative clinical risk reduction has not yet been demonstrated [23]. For example, EVs secreted by induced pluripotent stem cell-derived cardiac progenitor cells have been demonstrated to improve cardiac function in animal models of heart failure by delivering pro-angiogenic and anti-apoptotic microRNAs to injured myocardium. These EVs increased myocardial capillary density, reduced fibrosis, and enhanced ejection fraction in post-MI hearts [23]. The regenerative potential of EVs could be especially valuable in cardiac amyloidosis, where amyloid deposits cause irreversible myocardial damage. In theory, EVs from mesenchymal stem cells or cardiosphere-derived cells might help repair amyloid-injured myocardium by promoting angiogenesis, modulating fibrosis, and recruiting endogenous repair mechanisms [24]. Early support for this idea comes from general heart failure research: numerous preclinical studies have shown EV therapies improving cardiac function in models of myocardial infarction, myocarditis, and dilated cardiomyopathy by reducing inflammation, cell death, and fibrosis [25]. EV-based cardiac therapies consistently report reduced infarct sizes and enhanced cardiac function via EV-mediated delivery of beneficial signals [26].

Applied to amyloid cardiomyopathy, EV-based regenerative approaches might support myocardial recovery once the upstream amyloidogenic driver is controlled—after plasma cell-directed therapy in AL, or after stabilization or suppression of TTR production in ATTR (e.g., stabilizers or gene-silencing therapies) [27].

A novel therapy concept is to use EVs to interfere with amyloid formation or to promote amyloid clearance. For example, EVs could be loaded with amyloid-binding peptides or siRNAs that silence amyloid precursor proteins. The retro-inverso D-peptide mentioned earlier is one such approach; rather than injecting the peptide alone, future preparations might package it in EVs to improve delivery to cardiac tissue. In the Cimini et al. study, the D-peptide (DRI-5S) successfully prevented SAA3 monomers from aggregating in the heart, showing improved heart function post MI in murine models, but this has not been replicated in human trials as yet [29].

Finally, one emerging application is using EVs to deliver gene-silencing or gene-editing molecules [31]. This approach is particularly ATTR-relevant, since TTR is predominantly produced by the liver and is readily targetable by systemic delivery strategies. While current clinical trials use lipid nanoparticles to deliver siRNA or CRISPR to the liver, future strategies might employ engineered EVs derived from liver-tropic cells to carry gene silencers that selectively shut down mutant TTR synthesis [32]. EVs have natural tropism that can be exploited or enhanced by surface engineering, for example, displaying an antibody fragment or peptide that guides the EV to cardiac tissue or hepatic tissue. EV-based gene delivery could potentially reduce off-target effects and immunogenicity relative to synthetic nanoparticles, though this approach is still in development [33]. In contrast, analogous upstream EV-based approaches for AL would require targeting the plasma cell clone, and no AL-specific EV gene therapy strategies have yet entered clinical testing.

## 5. Limitations and Challenges

Despite these exciting prospects, significant challenges remain before EV therapeutics can become a clinical reality in amyloidosis (Table 2). First, the biodistribution and targeting of injected EVs is suboptimal; unmodified EVs often accumulate in clearance organs like the liver and spleen rather than the intended target (e.g., the heart). Efforts are underway to enhance cardiac delivery, such as conjugating homing ligands or using cardiac tissue engineering (e.g., hydrogel patches loaded with EVs) [34].

Second, there is a lack of universally accepted quality control and standardization procedures. Different isolation methods can yield EV products with varying purity, size distribution, and cargo profiles, and currently, there is no universally adopted protocol for EV manufacturing. This complicates dose standardization. EV dose could be quantified by particle number, protein content, or specific cargo molecule levels, but each measure has pitfalls if co-isolated contaminants are present.

Third, storage and stability issues require solutions: EVs may lose integrity or function upon long-term storage or freeze–thaw cycles. Cryopreservation at −80 °C in buffer can maintain EV stability, but this requires costly infrastructure and validation. Finally, rigorous safety evaluations are needed. The safety of EVs, though theoretically better than systemic therapies in cellular and animal studies, will need thorough assessment in human subjects before this can become a mainstay of treatment [22]. EVs are generally less immunogenic than whole cells, but they carry bioactive cargo that could have unintended effects. There is a theoretical risk of EVs delivering oncogenic signals or pro-fibrotic signals if not properly characterized. Thus, before EV therapy can enter the clinic for cardiac amyloidosis, researchers must address these translational challenges.

## 6. Clinical Implications, Biomarker Potential, and Future Directions

Currently, the clinical implementation of EV technologies is unclear, since this technology is still in its primordial stages. However, one of the most promising clinical applications of EVs lies in diagnostics and disease monitoring. Because EVs circulate in virtually all biofluids (blood, urine, saliva, etc.) and carry molecular signatures of their parent cells, they represent a readily accessible “liquid biopsy” of organ-specific processes [39]. In cardiac amyloidosis, where early diagnosis is crucial but often missed, EV-based biomarkers could significantly improve patient care. Recent studies support the feasibility of EV biomarkers in amyloid diseases.

### 6.1. AL Amyloidosis: EV-Associated Light-Chain Species in Urine and Blood

For instance, urinary EVs from patients with AL amyloidosis have been found to contain pathogenic light-chain oligomers-intermediates in fibril formation that are not present in patients with mere monoclonal gammopathy (MGUS) [40] showed that these urinary EV-borne light-chain oligomers could be detected and correlated with disease activity, raising the prospect of a non-invasive urine test to monitor renal amyloid burden and response to therapy [40]. This is especially relevant for AL amyloidosis with renal involvement, where invasive biopsies carry risk. However, while these data support EV-based detection of pathogenic light chain intermediates in AL, comparable EV biomarker studies focused specifically on cardiac involvement (rather than renal disease activity) remain limited.

### 6.2. ATTR Amyloidosis: Circulating EV Proteome and Tissue-Derived Injury Signatures

Similarly, work on ATTR amyloidosis suggests that analyzing circulating EV content might aid diagnosis. In a 2020 study, DiGiovanni et al. performed proteomic profiling of plasma EVs from ATTR cardiomyopathy patients. They discovered that EVs in ATTR patients were enriched with distinctive proteins, including cardiac-specific proteins (troponins, myosin fragments) and neuronal proteins, reflecting the multi-system nature of ATTR amyloidosis [41]. These EV proteomic signatures distinguished amyloid patients from controls and hinted at organ involvement (e.g., peripheral neuropathy in ATTR) through the presence of neuron-derived cargo in EVs. However, validation in larger cohorts and direct comparisons between ATTRwt and ATTRv EV signatures are still lacking. Ongoing research is rapidly expanding the catalogue of EV-based biomarkers. Efforts are underway to identify EV-contained miRNAs that change in cardiac amyloidosis, similar to how neuron-derived EV miRNAs are being studied in Alzheimer’s disease [42]. For example, EV miRs such as miR-21 and miR-146 have been implicated in cardiac fibrosis and inflammation. Similarly, amyloid heart may release EVs with a unique microRNA fingerprint indicative of stress or infiltrative cardiomyopathy [43]. Finally, it is highly plausible that EVs from cardiac amyloidosis patients could also have prognostic value, and some studies already support this for cardiovascular disease in general. The idea that baseline levels of EV-mediated injury signals correlate with patient outcomes is an active area of research, though more specific research is needed for cardiac amyloidosis [44,45]. However, it is important to stress that head-to-head studies comparing AL versus ATTR EV cargo (protein and RNA) in well-phenotyped cardiac amyloidosis cohorts are not yet available, representing a key translational gap.

EVs are increasingly being investigated in precision medicine and therapy. Lessons from oncology are particularly instructive—liquid biopsies analyzing extracellular vesicles and other circulating biomarkers have revolutionized personalized cancer treatment by enabling molecular profiling, early detection of resistance, and therapy optimization [30]. Similarly, in cardiac amyloidosis, several steps in the infiltrative and fibrotic pathways present potential targets for EV-based intervention. In fact, there is an ongoing clinical trial in France (SECRET-HF trial, NCT05774509, [28]) which is evaluating EVs derived from cardiac progenitor cells for heart failure secondary to non-ischemic cardiomyopathy. Participants receive repeated infusions (20–40 × 10^9^ particles/kg) of an EV-enriched secretome, with both biochemical and functional cardiac endpoints under evaluation. This study represents one of the first attempts to assess the safety and efficacy of systemically delivered EV-based therapeutics in human cardiac disease.

Despite the excitement, it is important to temper expectations: EV technology in the clinic is still in its infancy. To date, there are no approved EV-based diagnostics or therapeutics specifically for cardiac amyloidosis. The field faces practical issues such as establishing reference ranges for EV biomarkers and ensuring reproducible EV isolation from patient samples. Clinical trials will be needed to demonstrate that EV-guided decisions can indeed improve patient outcomes. As of 2025, no registered interventional trial is targeting amyloidosis with EV-based therapy (reflecting the complexity and novelty of this approach). However, the initiation of the SECRET-HF trial in dilated cardiomyopathy provides momentum for testing EV therapies in related conditions. It is reasonable to anticipate that within a few years, early-phase trials may emerge—for example, testing mesenchymal stem cell EV infusions in AL amyloidosis patients with cardiac involvement, to see if cardiac biomarkers improve or organ function stabilizes.

In conclusion, EVs are gaining a significant position in our understanding of cardiac amyloidosis. As described in this review, EVs can be double-edged swords, contributing to disease pathology on one hand, yet offering new opportunities for diagnosis and therapy on the other. For clinicians and researchers, EVs could link molecular mechanisms to clinical practice and provide an opportunity to disrupt and prevent amyloidosis at the level of intercellular crosstalk. Future research should focus on bridging the gap between bench and bedside, standardizing EV measurement techniques, validating EV-based biomarkers in multicenter cohorts, and conducting more EV-based clinical trials. The hope is that EV-centered strategies will help with the management of cardiac amyloidosis, leading to earlier detection, personalized therapies, and improved outcomes for this challenging disease.

## Figures and Tables

**Table 1:** Therapeutic Strategies Using EVs Relevant to Cardiac Amyloidosis.

Strategy	Source/Approach	Mechanism	Target Pathway	Delivery/Targeting	Evidence Level	Current Status	Key References
Stem-cell or progenitor-derived EVs	Cardiac progenitor, mesenchymal, iPSC	Promote angiogenesis, reduce fibrosis, restore function	Fibrosis, remodeling, repair	Intravenous infusion	Preclinical, Phase I (SECRET-HF 2024)	Safe, early efficacy	[22,27,28]
EV-based amyloid antagonists	Engineered EVs w/D-peptide or siRNA	Inhibit SAA3 or TTR aggregation	SAA/TTR aggregation	Systemic (experimental IV)	Preclinical (evidence derived from mouse models)	Experimental	[29]
EV-based gene therapy	Liver-targeted EVs w/CRISPR or siRNA	Silence mutant TTR synthesis	TTR production	Liver-tropic EVs/ligand-targeted	Preclinical concept	No human data	[22,27]
EV-mediated immune modulation	Regulatory immune-cell EVs	Reduce inflammation and fibrosis	Immune/fibrosis	Intravenous	Conceptual	Not yet tested	[30]
EV-loaded cardioprotective drugs	Engineered EVs	Targeted drug delivery to myocardium	Remodeling, ischemia	IV/hydrogel patch targeted	Preclinical	Optimization ongoing	[9,11]

Abbreviations: iPSC: induced pluripotent stem cell; siRNA: small interfering RNA; CRISPR: clustered regularly interspaced short palindromic repeats; IV: intravenous; EVs: extracellular vesicles; TTR: transthyretin; SAA: serum amyloid A.

**Table 2:** Key Translational limitations and Solutions.

Limitation	Impact	Emerging Solution	Reference
Inconsistent EV isolation/reporting	Reproducibility issues	MISEV-2023 guidelines application, EV-TRACK, harmonized reporting	[35,36]
Quantification variability	Dosing uncertainty	Multi-metric quantification (particles + protein + RNA)	[35]
Off-target biodistribution	Reduced cardiac delivery and off-target effects	Cardio-tropic ligand engineering, local delivery hydrogels	[9,22]
GMP scale-up and QC	Regulatory bottleneck	Closed-system bioreactors, potency assays	[22,27]
Storage degradation	Loss of integrity and function	Optimized cryoprotectants, lyophilization	[35]
Safety/immunogenicity	Off-target effects/toxicity/Clinical risk	Standardized preclinical toxicology and CQA testing	EMA ATMP guideline (2024 consultation; Section 6) [37] FDA draft guidance (December 2023): Potency Assurance for CGT products (pp. 5–7, [38])
Lack of amyloidosis-specific data	Limited clinical validation	Multicenter observational and interventional EV studies	[16,29]

## Data Availability

No new data were created or analyzed in this study. Data sharing is not applicable to this article.

## Abbreviations

EVs: extracellular vesicles; TTR: transthyretin; SAA: serum amyloid A; siRNA: small interfering RNA; MSC: mesenchymal stem cells.
